# Involvement of Sulfur in the Biosynthesis of Essential Metabolites in Pathogenic Fungi of Animals, Particularly *Aspergillus* spp.: Molecular and Therapeutic Implications

**DOI:** 10.3389/fmicb.2019.02859

**Published:** 2019-12-13

**Authors:** Aimee M. Traynor, Kevin J. Sheridan, Gary W. Jones, José A. Calera, Sean Doyle

**Affiliations:** ^1^Department of Biology, Maynooth University, Maynooth, Ireland; ^2^Centre for Biomedical Science Research, School of Clinical and Applied Sciences, Leeds Beckett University, Leeds, United Kingdom; ^3^Instituto de Biología Funcional y Genómica (IBFG-CSIC), Universidad de Salamanca, Salamanca, Spain; ^4^Departamento de Microbiología y Genética, Universidad de Salamanca, Salamanca, Spain

**Keywords:** *Aspergillus*, sulfur, ergothioneine, gliotoxin, zinc

## Abstract

Fungal sulfur uptake is required for incorporation into the sidechains of the amino acids cysteine and methionine, and is also essential for the biosynthesis of the antioxidant glutathione (GSH), *S*-adenosylmethionine (SAM), the key source of methyl groups in cellular transmethylation reactions, and *S*-adenosylhomocysteine (SAH). Biosynthesis of redox-active gliotoxin in the opportunistic fungal pathogen *Aspergillus fumigatus* has been elucidated over the past 10 years. Some fungi which produce gliotoxin-like molecular species have undergone unexpected molecular rewiring to accommodate this high-risk biosynthetic process. Specific disruption of gliotoxin biosynthesis, via deletion of *gliK*, which encodes a γ-glutamyl cyclotransferase, leads to elevated intracellular antioxidant, ergothioneine (EGT), levels, and confirms crosstalk between the biosynthesis of both sulfur-containing moieties. Gliotoxin is ultimately formed by gliotoxin oxidoreductase GliT-mediated oxidation of dithiol gliotoxin (DTG). In fact, DTG is a substrate for both GliT and a *bis*-thiomethyltransferase, GtmA. GtmA converts DTG to bisdethiobis(methylthio)gliotoxin (BmGT), using 2 mol SAM and resultant SAH must be re-converted to SAM via the action of the Methyl/Met cycle. In the absence of GliT, DTG fluxes via GtmA to BmGT, which results in both SAM depletion and SAH overproduction. Thus, the negative regulation of gliotoxin biosynthesis via GtmA must be counter-balanced by GliT activity to avoid Methyl/Met cycle dysregulation, SAM depletion and *trans* consequences on global cellular biochemistry in *A. fumigatus*. DTG also possesses potent Zn^2+^ chelation properties which positions this sulfur-containing metabolite as a putative component of the Zn^2+^ homeostasis system within fungi. EGT plays an essential role in high-level redox homeostasis and its presence requires significant consideration in future oxidative stress studies in pathogenic filamentous fungi. In certain filamentous fungi, sulfur is additionally indirectly required for the formation of EGT and the disulfide-bridge containing non-ribosomal peptide, gliotoxin, and related epipolythiodioxopiperazines. Ultimately, interference with emerging sulfur metabolite functionality may represent a new strategy for antifungal drug development.

## Introduction

Sulfur is an essential element that is incorporated into various cellular metabolites and used for multiple metabolic processes in fungi, including the proteogenic amino acids cysteine (Cys) and methionine (Met), oxidative stress defense [glutathione (GSH) and ergothioneine (EGT)], methylation [*S*-adenosylmethionine (SAM)], epipolythiodioxopiperazine (ETP) toxin biosynthesis (e.g., gliotoxin) and iron metabolism (Fe–S clusters) (Davis et al., 2011; Struck et al., 2012; Amich et al., 2013; Sheridan et al., 2016). Fungi, such as the opportunistic pathogen *Aspergillus fumigatus*, can acquire sulfur either from inorganic or organic sources (Amich et al., 2013). In *A. fumigatus*, sulfur availability is an important consideration for pathogenesis, as it must be sourced from the environment for fungal survival (Amich et al., 2016). Due to the differences between human and fungal sulfur assimilatory pathways, the enzymes and intermediates produced by fungi represent attractive drug targets to potentially enable treatment of these serious infections. It is also important to note that interplay occurs between many components of these sulfur metabolic pathways with other systems in pathogenic fungi (Yadav et al., 2011). These also present opportunities for antifungal drug target development. In addition to *A. fumigatus*, the most common human pathogenic fungi are *Candida* and *Cryptococcus* spp., and these opportunistic yeast species also possess sulfur uptake and utilization pathways which are often essential for virulence. Hydroxyl radicals can also be dissipated via cellular thioredoxin/thioredoxin reductases (TRR), and glutaredoxin/glutathione reductases, respectively, as elegantly reviewed in Zhang and Feng (2018). Moreover, TRR inhibitors have been proposed as selective antifungal drugs to target *A. fumigatus*, and TRR also contributes to fungal pathogenicity in *Fusarium graminearum* (Fan et al., 2019; Marshall et al., 2019).

This review initially focuses on sulfur assimilation systems, the biosynthesis of established sulfur-containing metabolites (Cys, Met, GSH, and SAM) and highlights potential antifungal drug targets. It then proceeds to describe the metabolic consequences of aberrant cellular methylation, and after introducing advances in the biosynthesis and self-protection against gliotoxin (Dolan et al., 2015), attempts to illustrate how new thinking on the apparent integration of this biosynthetic pathway with SAM homeostasis (Owens et al., 2015) might be exploited therapeutically. The recent demonstration of interplay between gliotoxin biosynthesis and zinc homeostasis (Saleh et al., 2018; Vicentefranqueira et al., 2018) is then elaborated and presented in a context which serves to illustrate how this unprecedented finding may prove useful in future mechanistic studies pertaining to fungal virulence. Disruption of gliotoxin biosynthesis led to the discovery of EGT in *A. fumigatus*, and the review then pivots to describe the biochemistry and functionality of this high level antioxidant in fungi (Gallagher et al., 2012). Indeed, we contend that the discovery of EGT may necessitate re-consideration of previous studies pertaining to the oxidative stress sensitivity of *A. fumigatus*, and possibly other fungi, following deletion of reactive oxygen species (ROS)-dissipating systems (Lessing et al., 2007; Lambou et al., 2010). We then move to reveal and illustrate that both gliotoxin and EGT biosynthesis (Sheridan et al., 2016; Dolan et al., 2017) has led to molecular rewiring in *A. fumigatus*, whereby Cys, GSH and SAM have become substrates for the biosynthesis of the aforementioned metabolites, along with playing a role in gliotoxin ‘activation’ and attenuation, leading to unexpected consequences for fungal metabolic systems (Owens et al., 2015). Finally, we introduce the concept of a ‘cystathionine switch’ in *A. fumigatus* which may play a role in fluxing thiol-containing metabolites toward different fates, depending on the prevailing cellular redox homeostatic requirements (Sheridan et al., 2016). Overall, we attempt to paint a highly dynamic and integrated ‘thiometabolic’ system in fungi, which if disrupted, may have unforeseen potential for new antifungal drug development.

### Sulfur Assimilation

**FIGURE 1:**
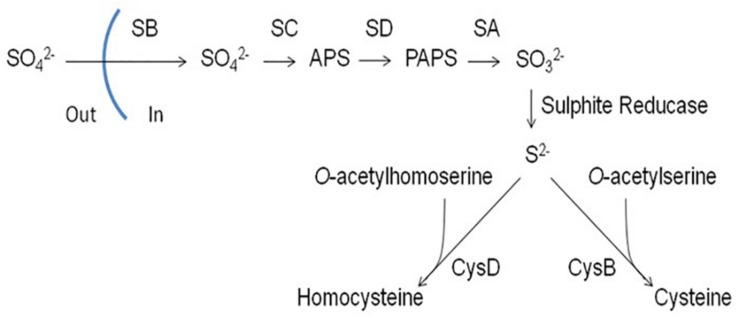
Assimilation of inorganic sulfur (sulfate) in filamentous fungi. *SB*, sulfate permease; *SC*, ATP sulphurylase; *SD*, adenosine 5′-phosphosulphate (APS) kinase; *SA*, 3′-phosphoadenosine-5′-phosphosulphate (PAPS) reductase; *CysB*, cysteine synthase; *CysD*, homocysteine synthase (Amich et al., 2013).

Inorganic sources of sulfur that can be utilized by filamentous fungi include sulfate (SO_4_^2–^), sulfite (SO_3_^2–^) and sulfide (S^2–^) (Brzywczy and Paszewski, 1994; Paszewski et al., 1994). Sulfate is widespread in nature and its assimilation pathway has been well-characterized in filamentous fungi. Sulfate is first transported into the cell by a sulfate permease (SB). Following transport into the cell, sulfate is reduced to adenosine 5′-phosphosulfate (APS) by an ATP sulfurylase (SC). APS is then phosphorylated by an APS kinase (SD) to form 3′-phosphoadenosine 5′-phosphosulfate (PAPS) followed by sulfite release from PAPS via the PAPS reductase SA. Sulfite is reduced to sulfide by a sulfite reductase (AFUA_6G08920, α-subunit; SF: β-subunit) which can then be incorporated into Cys via *O-*acetylserine through a process catalyzed by CysB (a cysteine synthase) or homocysteine (Hcy) via *O*-acetylhomoserine through a process catalyzed by CysD (a homocysteine synthase) (Figure 1) (Marzluf, 1997).

It has been demonstrated that SB is essential for sulfate assimilation in *A. fumigatus* (Amich et al., 2013). Deletion of *sB* resulted in abrogation of growth on media containing sulfate as a sole sulfur source. This confirmed that SB functions in *A. fumigatus* as a sulfate permease and is non-redundant. Δ*sB* also showed reduced growth on sulfite and thiosulfite, indicating that SB contributes to the transport of other inorganic sulfur sources. Amich et al. (2013) also studied the sulfite reductase responsible for converting sulfite into sulfide whereby the gene encoding the β subunit of sulfite reductase, *sF*, was deleted. Δ*sF* was incapable of growing on any inorganic sulfur source, with the exception of sulfide. Sulfite reductase is therefore essential for assimilation of inorganic sulfur sources, apart for sulfide.

MetR is a leucine zipper (bZIP) transcription factor and orthologs in *A. nidulans* (MetR) and *N. crassa* (CYS-3) have been demonstrated to regulate sulfur assimilation in their respective organisms (Fu et al., 1989; Natorff et al., 2003). MetR has also been demonstrated to regulate inorganic sulfur assimilation in *A. fumigatus* (Amich et al., 2013) where deletion of *metR* resulted in a complete absence of growth on media with inorganic sulfur as a sole sulfur source. Under sulfur rich conditions, MetR was demonstrated to be uniformly distributed throughout the cytoplasm, however, under sulfur-depleted conditions, MetR localized strongly to the nucleus. MetR localisation to the nucleus was followed by upregulation of genes involved in the inorganic sulfur assimilation pathway: *sB, sC, sD, sA* and sulfite reductase. MetR was also demonstrated to bind to the promoter regions of *sB, sC*, and *sD.* Deletion of *metR* resulted in decreased transcription of *sB, sC, sD, sA*, and sulfite reductase relative to wild-type levels. MetR was therefore demonstrated to regulate inorganic sulfur assimilation (Amich et al., 2013).

*Aspergillus fumigatus* can also utilize organic sulfur sources such as Met, Cys, Hcy and taurine (a cysteine derivative), however, regulation of the assimilation of organic sources is independent of MetR. Interestingly, *A. fumigatus* wild-type and Δ*metR* showed equivalent growth on Met- or Hcy-containing media as sole sulfur sources (Amich et al., 2013). Conversely, Δ*metR* showed severely reduced growth on Cys-containing media as a sole sulfur source, compared to wild-type, and could not grow on media with either GSH or taurine as a sole sulfur source. However, it was noted that Δ*metR* could utilize GSH and Cys as a sole sulfur source when simultaneously subjected to nitrogen starvation, indicating a link between sulfur and nitrogen utilization. Taurine could not be utilized by Δ*metR* regardless of nitrogen availability. Amich et al. (2016) subsequently demonstrated that sulfite reductase is also required for taurine assimilation since taurine catabolism releases sulfite, which is reduced to sulfide via sulfite reductase. Since sulfite reductase is under MetR regulation, it follows that taurine cannot be utilized as a sulfur source in Δ*metR*.

Analysis of genes involved in Met uptake and catabolism revealed a somewhat inconsistent expression dependency on MetR. For example, expression analysis of genes encoding putative Met uptake proteins *mupA* and *mupC* revealed that *mupC* expression was not altered in Δ*metR*, while *mupA* expression was reduced in Δ*metR* compared to wild-type. *metAT*, encoding a methionine aminotransferase, showed increased expression in Δ*metR* compared to wild-type under sulfur-limiting conditions. *metAT* is predicted to effect Met catabolism, and to be essential for utilization of Met as a sulfur source. Upregulation of *metAT* in Δ*metR* under sulfur-limiting conditions indicates that not only is Met utilization independent of MetR, but can be increased in order to compensate for loss of inorganic sulfur utilization. Cys utilization could not be properly addressed as the mechanisms of Cys catabolism have not been fully elucidated. However, *cysA*, encoding a putative cysteine permease, and *cysD* (cysteine synthase) appear to be regulated in a MetR-dependant manner, indicating that Cys uptake and assimilation may be regulated by MetR (Amich et al., 2013).

The importance of efficient sulfur utilization in fungal virulence was demonstrated for *A. fumigatu*s Δ*metR*, which showed decreased virulence in *Galleria mellonella* compared to wild-type, indicating that sulfur assimilation can be a limiting factor in the pathogenesis of *A. fumigatus* (Amich et al., 2013). Deletion of *sB* and *sF* in *A. fumigatus* did not result in reduced virulence in a murine model, which indicated that neither inorganic sulfur sources or taurine serve as essential sulfur sources during infection. Furthermore, a Cys auxotroph mutant displayed reduced virulence in a murine model, indicating that Cys levels in the murine lung are insufficient to act as a sulfur source during infection (Amich et al., 2016). While MetR positively regulates sulfur assimilation, 4 genes (*sconA, sconB, sconC*, and *sconD*) act to negatively regulate sulfur assimilation in filamentous fungi. In *A. nidulans*, deletion of *scon* genes results in excessive accumulation of sulfur-containing amino acids. Notably, exposure to Met had no effect on sulfate assimilation in *scon* deletion mutants, while in wild-type, Met exposure almost eliminated inorganic sulfur assimilation (Natorff et al., 1993). *metR* transcription increases in *scon* deletion mutants, particularly *sconB2*, indicating interaction between the positive and negative elements of sulfur regulation (Natorff et al., 2003).

*Candida glabrata* and *Candida albicans* both have very similar sulfur assimilatory pathways to that of the model yeast *Saccharomyces cerevisiae*. In yeast, *MET15* encodes the enzyme *O*-acetylhomoserine *O*-acetylserine sulfydrylase which is responsible for the production of Hcy and Cys. *MET15* deletion from *S. cerevisiae* and *C. glabrata* resulted in organic sulfur auxotrophy, whereas *C. albicans* Δ*met15* was able to grow on inorganic sulfate as a sulfur source (Viaene et al., 2000). When GSH biosynthesis was abrogated in these pathogenic yeasts, a requirement for GSH sequestration from the host, or other organic sources of sulfur, ensues in order to survive. It has been shown that *C. albicans* Δ*met15* is capable of utilizing exogenous GSH for growth through the presence of high-affinity GSH transporters and a functional degradation pathway (DUG). However, the absence of a functional endogenous GSH production pathway resulted in attenuated virulence of *C. albicans* in the host. This suggests that the amount of GSH in plasma is insufficient to provide the levels required to protect the yeast against the high levels of redox stress from the host (Desai et al., 2011). Conversely, *C. glabrata* Δ*met15* is unable to utilize exogenous GSH due to the absence of a GSH transporter. It does, however, have a functional DUG pathway for utilization of intracellular GSH. Both *C. albicans* and *C. glabrata* Δ*met15* are capable of growth on Met, Cys and cystine as organic sulfur sources, suggesting the presence of forward and reverse transsulfuration mechanisms in these yeasts (Yadav et al., 2011). Both *C. albicans* and *C. glabrata* Δ*met15* mutants exhibit reduced virulence, underlying the importance of sulfur metabolism for pathogen function in the host.

### Biosynthesis of Sulfur-Containing Amino Acids and Metabolites

**FIGURE 2:**
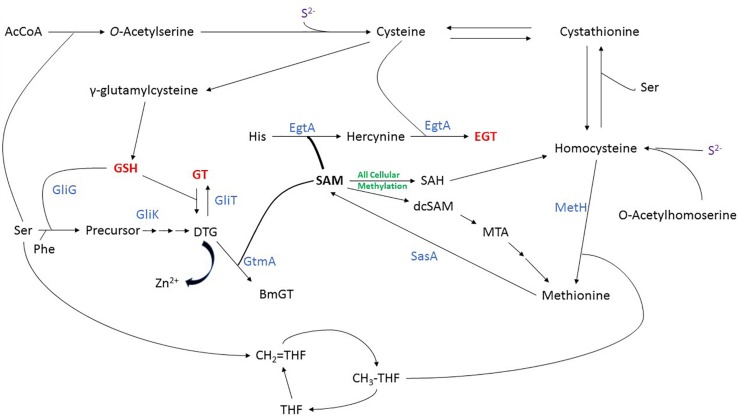
Integrated sulfur metabolism in *A. fumigatus*, demonstrating the biosynthesis of sulfur-containing amino acids and metabolites, especially gliotoxin, BmGT, dithiol gliotoxin (DTG), ergothioneine (EGT) and GSH. Nascent evidence of interaction between DTG and Zn^2+^ is also noted. Enzymes are shown in blue.

Cys can be formed directly from assimilated inorganic sulfur, using cysteine synthase (CysB) which converts sulfide and *O*-acetylserine into cysteine. Met can be formed from inorganic sulfur via Hcy whereby Hcy synthase (CysD) catalyzes the formation of Hcy from sulfide and *O*-acetylhomoserine. A cobalamin-independent methionine synthase (MetH) then catalyzes the transfer of a methyl group from methyltetrahydrofolate to Hcy to form Met (Figure 2) (Brzywczy et al., 2007; Saint-Macary et al., 2015). Importantly (Amich et al., 2016) revealed that MetH is essential for the virulence of *A. fumigatus* which highlights Met biosynthesis as a potential target for antifungal drug development. Cys and Hcy can be interconverted through the transsulfuration pathway, via the intermediate cystathionine. The forward transsulfuration pathway, catalysed by cystathionine γ-synthase (CGS; MetB), converts Cys into cystathionine. Cystathionine is then converted into Hcy by cystathionine β-lyase (CBL; MetG). The reverse transsulfuration pathway sees Hcy converted into cystathionine by cystathionine β-synthase (CBS; MecA) and cystathionine converted into Cys by cystathionine γ-lyase (CGL; MecB). Through this pathway, thiol groups can be transferred between Cys and Hcy (Figure 2) (Sienko et al., 2009; Sohn et al., 2014). Furthermore, Met and Hcy are two components of the “Met cycle,” which also includes SAM and *S*-adenosylhomocysteine (SAH). In *A. nidulans*, Met is converted to SAM by a single copy SAM synthetase (SasA), which is essential for fungal growth (Gerke et al., 2012). SAM is the universal methyl donor (Struck et al., 2012), and donation of the methyl group from SAM leads to the formation of SAH. SAH can then be converted into Hcy via *S*-adenosylhomocysteinase (Sah1) catalyzed hydrolysis. As before, Hcy is converted to Met by methionine synthase (MetH) to complete the cycle (Figure 2) (Owens et al., 2015; Saint-Macary et al., 2015). Met can be also recovered from SAM via the Met salvage cycle. In the Met salvage pathway, SAM is decarboxylated to form decarboxylated SAM (dcSAM). In a reaction catalyzed by spermidine synthase, dcSAM donates aminopropyl groups to putrescine to form spermidine and 5′-methylthioadenosine (MTA). MTA is then converted through a series of intermediates back to Met (Sauter et al., 2013). This pathway also contributes to the regulation of cellular Met and SAM homeostasis in fungi (Figure 2).

The presence of the opportunistic yeast pathogen *Cryptococcus neoformans* is asymptomatic in immunocompetent individuals, yet causes serious disease in immunocompromised patients. Sulfur uptake and biosynthesis of Met and Cys have previously been shown to modulate survival of *C. neoformans* in the host, where the interlinked pathways of sulfur uptake and amino acid biosynthesis exhibits forward and reverse transsulfuration pathways in this yeast (Yang et al., 2002). Studies have focused on Δ*met2*, Δ*met3* and Δ*met6* knockouts of *C. neoformans* to investigate their effects not only on metabolism but also virulence of this pathogen. MET6, responsible for synthesizing Met from Hcy, was shown to be transcriptionally regulated by the presence of exogenous Met and Hcy. In fact, *C. neoformans*Δ*met6* and Δ*met3* are both Met auxotrophs, however, the Δ*met6* mutant was unable to satisfy this auxotrophy with Cys or Hcy as the sole sulfur source while the Δ*met3* could (Pascon et al., 2004). It was suggested that this difference in growth of the two mutants was caused by a build-up of Hcy in Δ*met6*, which may impact on other important pathways such as ergosterol biosynthesis. Similarly to *MET3* and *MET6*, *MET2* is also necessary for Met biosynthesis in *C. neoformans* and disruption of this gene leads to Met auxotrophy and avirulence (Nazi et al., 2007). Further, it has been revealed that *CYS3* encodes a transcriptional activator that upon deletion causes a Met/Cys auxotrophy and that Cys3 is the major regulator of inorganic sulfur uptake in *C*. *neoformans* (de Melo et al., 2019).

It is important to note, however, that the fungal transsulfuration pathway, the Methyl/Met cycle and the Met salvage pathway have primarily been elucidated in *Saccharomyces cerevisiae* and *A. nidulans*, both of which are devoid of the *gli* biosynthetic gene cluster which encodes gliotoxin biosynthesis and bisdethiobis(methylthio)gliotoxin (BmGT) formation – which are highly dependent on SAM availability (Dolan et al., 2014; Manzanares-Miralles et al., 2016; Smith et al., 2016). Furthermore, the critical role of SAM in the biosynthesis of ergothioneine (EGT) in fungi has only recently been appreciated (Jones et al., 2014; Sheridan et al., 2016). Indeed, it is now also abundantly clear that Cys provides the thiol group for two important redox active metabolites in *A. fumigatus*: GSH (directly) and gliotoxin (indirectly) (Davis et al., 2011; Scharf et al., 2011). Cys is converted to GSH via a two-step pathway in fungi (Schmacht et al., 2017). The first step is likely catalyzed by glutamate–cysteine ligase (Gcs1), which catalyzes the condensation of Cys and glutamate to form γ-glutamylcysteine, which is then converted to GSH by the addition of Gly to the C-terminal of γ-glutamylcysteine by glutathione synthase (Gsh2) (Chen et al., 2009). Moreover, both Cys and pantothenic acid are key precursors of the thiol-containing Coenzyme A in yeast, and in other fungi (Chiu et al., 2019). CoA is not only an important co-factor for enzyme-catalyzed reactions, but is *the* source of 4′-phosphopantetheine for essential post-translational activation of non-ribosomal peptide synthetases and polyketide synthases in fungi (Stack et al., 2009; Sheridan et al., 2015). Furthermore, it has been shown that disruption of pantothenic acid biosynthesis, via *panA* deletion in *A. fumigatus*, resulted in a mutant strongly attenuated for virulence in a number of immunocompromised mouse models (Dietl et al., 2018).

### SAM, SAH and Methylation

Functionally, methylation is involved in signal transduction, protein repair, chromatin regulation, gene silencing and secondary metabolite biosynthesis (Schubert et al., 2003) (Liscombe et al., 2012; Sarikaya-Bayram et al., 2014). SAM provides the methyl group for the majority of cellular methylation reactions, across all branches of life, and as such is known as the universal methyl donor (Struck et al., 2012). As noted, donation of the methyl group from SAM results in the formation of SAH (Figure 2). Relevantly, SAH is a potent inhibitor of methylation (Caudill et al., 2001), and dissipation of SAH is important to avoid impaired cellular methylation, otherwise elevated SAH may impede mycelial growth. Consequently, SAM/SAH is considered a useful indicator of the cellular methylation potential, and perturbation of SAM/SAH has been studied through the disruption of *S*-adenosylhomocysteinase activity (Liao et al., 2012). As *S*-adenosylhomocysteinase catalyses the hydrolysis of SAH, any disruption to its activity would be expected to increase SAH levels, and reduce the SAM/SAH ratio. This would therefore be expected to inhibit methylation (Caudill et al., 2001). It has also been demonstrated that inhibition of *S*-adenosylhomocysteinase using 9-(S)-(2,3-dihydroxypropyl)-adenine (DHPA) in tobacco seeds resulted in global hypomethylation of DNA (Fulnecek et al., 2011). Phenotypically, this resulted in dosage-dependant developmental defects, linked to increased expression of floral organ identity genes. The authors concluded that tobacco seeds were particularly sensitive to accurate SAM/SAH levels at critical developmental periods. In fungi, Liao et al. (2012) investigated the effect of perturbed SAM/SAH in *Cryphonectria parasitica*. Deletion of the gene encoding *S*-adenosylhomocysteinase resulted in almost 10-fold increase in SAH abundance, and a twofold increase in SAM levels. This reduced the intracellular SAM/SAH from 2.63 to 0.53 and resulted in reduced radial growth, decreased asexual development and loss of pigmentation, proposed to be due to a decrease in DNA methylation. Regulation of intracellular SAM/SAH levels is therefore critical, as disruption can adversely affect tightly regulated gene expression, and potentially impede cell growth. How this is achieved in fungi capable of gliotoxin, BmGT and EGT biosynthesis (Cramer et al., 2006; Dolan et al., 2014; Sheridan et al., 2016), all of which are highly dependent on SAM utilization, and concomitant SAH generation (Owens et al., 2015), is only just becoming apparent – and potentially represents a new direction in the search for antifungal drug targets.

### Gliotoxin Biosynthesis

**FIGURE 3:**
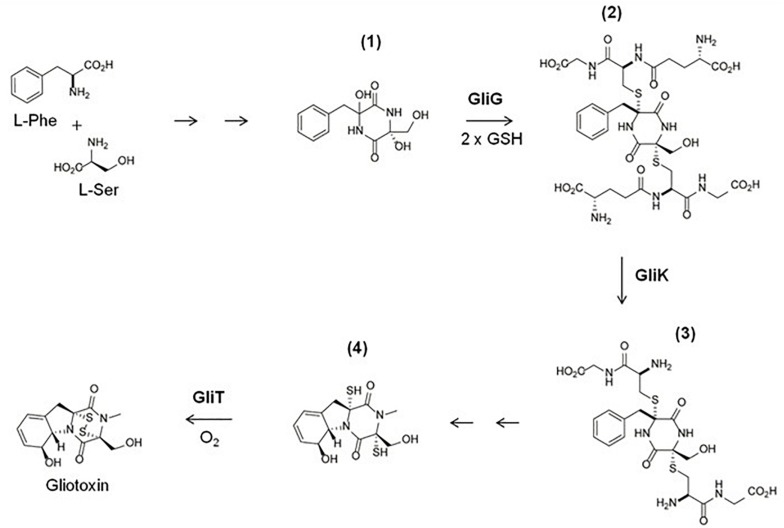
An abridged schematic of the gliotoxin biosynthetic pathway, demonstrating GliG, GliK, and GliT activity. L-Phe and L-Ser are processed by GliP and GliC to form intermediate **(1)**. **(1)** Is then doubly thiolated using 2 GSH molecules by GliG, to form a sulphurised intermediate **(2)**. **(2)** Then undergoes *bis*-glutamyl elimination, catalyzed by GliK, to yield **(3)**. **(3)** Then undergoes several more enzymatic reactions to yield **(4)**. The final product, gliotoxin, is then formed from **(4)** by the closure of the disulfide bridge via GliT (Schrettl et al., 2010; Gallagher et al., 2012; Scharf et al., 2013).

*De novo* gliotoxin biosynthesis occurs as follows: Phenylalanine and serine are condensed by the non-ribosomal peptide synthetase GliP (Balibar and Walsh, 2006) and monooxygenase GliC (Chang et al., 2013) to form intermediate **(1)** (Figure 3). Intermediate **(1)** is then doubly thiolated by the glutathione *S*-transferase GliG, using *two* GSH molecules, to form a *bis*-sulfurised intermediate **(2)** (Davis et al., 2011; Scharf et al., 2011). **(2)** Then undergoes *bis*-glutamyl elimination, catalyzed by γ-glutamyl cyclotransferase GliK (Gallagher et al., 2012), to yield **(3),** which then undergoes further processing reactions to yield **(4)**, dithiol gliotoxin (DTG) (Scharf et al., 2013). The final product, gliotoxin, is then formed from DTG by the closure of the disulfide bridge via GliT (Scharf et al., 2010; Schrettl et al., 2010; Dolan et al., 2017). Davis et al. (2011) identified a methylated shunt product, potentially generated by methyltransferase GliM. GliN was shown to effect SAM-dependent *N*-methylation (Scharf et al., 2014), and Dolan et al. (2014, 2017), Scharf et al. (2014), and Manzanares-Miralles et al. (2016) revealed SAM-dependency of BmGT formation catalyzed by gliotoxin thiomethyltransferase [GtmA (termed TmtA in Scharf et al., 2014)]. This methyltransferase was also shown by us to *sequentially* convert DTG to a *bis*-thiomethylated derivative, BmGT, via a monomethyl gliotoxin (MmGT) intermediate (Dolan et al., 2014, 2017; Manzanares-Miralles et al., 2016). Thus, during gliotoxin and BmGT biosynthesis, both thiol groups originate from two GSH moieties, and SAM is also a key provider of 2 – 4 methyl groups (Figures 2, 3). This inextricably links gliotoxin/BmGT formation to the cellular Methyl/Met cycle via GtmA (Owens et al., 2015) (Figure 2) and leads to the conclusion that biosynthesis and dissipation of the disulfide-containing non-ribosomal peptide gliotoxin requires significant utilization of cellular sulfur-containing metabolites, GSH and SAM (Figure 2).

Undoubtedly, the GliT-mediated self-resistance mechanism (Schrettl et al., 2010; Scharf et al., 2010) against gliotoxin in *A. fumigatus*, which has emerged as a predicate for analogous systems in other ETP-producing fungi, represents one of the most interesting aspects of gliotoxin biology. In essence, the initial observation that exogenously added gliotoxin altered the abundance of 22 proteins in *A. fumigatus* wild-type, including that of GliT, gliotoxin oxidoreductase, raised speculation as to the functionality of this enzyme (Schrettl et al., 2010). Indeed, it is now known that GliT abundance increases up to 75-fold upon *A. fumigatus* exposure to gliotoxin (Owens et al., 2015). Deletion of *gliT* rendered *A. fumigatus* highly sensitive to exogenous gliotoxin and also abolished gliotoxin biosynthesis (Schrettl et al., 2010; Scharf et al., 2010). While *A. fumigatus ΔgliT* growth was significantly impeded by exogenous gliotoxin, the mutant grew as per wild-type in gliotoxin *absence*. Importantly, *gliT* reconstitution in *A. fumigatus ΔgliT* restored both gliotoxin self-resistance and production. Unusually, *gliT* expression was shown to be independent of the overall *gli* cluster, as it could be specifically induced in *A. fumigatus ΔgliZ* (Bok et al., 2006) by gliotoxin addition (Schrettl et al., 2010). At the time, concern was expressed that the dense arrangement of genes in the *gli* cluster raised some uncertainty about the specific role of *gliT* in mediating self-protection against gliotoxin, however, Schrettl et al. (2010) uniquely showed that deletion of either *gliF* or *gliH*, both adjacent to *gliT*, did not result in acquisition of gliotoxin sensitivity. Thus, these authors unambiguously confirmed the unique role of GliT in *A. fumigatus* as *the gli* cluster-encoded, self-protection enzyme, against the reactive non-ribosomal peptide, gliotoxin (Schrettl et al., 2010).

Subsequent work revealed that GSH contributed to gliotoxin sensitivity, since diamide, a GSH scavenger, was less toxic to *A. fumigatus ΔgliT* compared to wild-type, which aligned with elevated GSH detected in the deletion strain (Carberry et al., 2012). Moreover, increased resistance to exogenous gliotoxin was seen for *S. cerevisiae* Δ*GSH1*, which contains significantly lower levels of intracellular GSH compared to wild-type yeast, and a yeast strain engineered to express *gliT* also became more resistant to gliotoxin. Thus, as is the case in animal cells (Bernardo et al., 2003), it is now thought that fungal uptake of gliotoxin results in GSH-mediated chemical reduction of the gliotoxin disulfide to yield DTG, which not only can lead to GSH depletion but strongly suggests that gliotoxin-mediated intracellular cytotoxicity is dependent on GSH presence.

It has also been revealed in *A. fumigatus* that GliT mediates DTG conversion to gliotoxin which is then effluxed from mycelia via GliA, an MFS transporter (Owens et al., 2015). Deletion of GliA abolished gliotoxin efflux and led to alternative secretion of BmGT. Thus, if gliotoxin cannot be made from DTG, or DTG is overproduced, GtmA effects SAM-dependent *bis*-thiomethylation of DTG to form BmGT (Dolan et al., 2014; Owens et al., 2015). This ultimately switches off gliotoxin biosynthesis, since the *gli* cluster requires DTG/gliotoxin for continued expression. Thus, no GliT means no DTG efflux (as gliotoxin), and detrimental intracellular DTG accumulation, allied to potential disruption of SAM/SAH due to enhanced GtmA activity for BmGT formation (Dolan et al., 2014; Owens et al., 2015). Although GtmA effectively dissipates DTG in *A. fumigatus*, using 2 mol SAM in the process, deletion of *gtmA* does not result in acquired gliotoxin sensitivity but instead causes overproduction of gliotoxin coincident with elevated abundance of the gliotoxin biosynthetic enzymes GliP and GliM (Dolan et al., 2014). This observation led us to propose that the primary GtmA function is to negatively regulate gliotoxin biosynthesis, post-DTG formation- although *gtmA* orthologs may be protective against gliotoxin in non-gliotoxin producing fungal species (e.g., *Aspergillus niger*) (Manzanares-Miralles et al., 2016). Overall, quantitative proteomic analysis, and parallel RNAseq (O’Keeffe et al., 2014), allied to intracellular metabolite quantification, has uncovered hitherto unforeseen systems interactions between gliotoxin/BmGT biosynthesis, self-resistance, sulfur metabolism and potentially altered Zn^2+^ availability (Dolan et al., 2017; Doyle et al., 2018; Saleh et al., 2018) in *A. fumigatus*.

Overall, these findings lead to the conclusion that GliT not only catalyzes formation of gliotoxin, but simultaneously prevents the dysregulation SAM/SAH and consequential deleterious impacts of elevated SAH, production of toxin Hcy and depletion of Met (Owens et al., 2015). In other words, while GtmA links BmGT formation to the Methyl/Met cycle, it appears that GliT is the enzyme which not only mediates, but *facilitates* gliotoxin biosynthesis without adversely affecting sulfur metabolite homeostasis in *A. fumigatus*. So, although *bis*-thiomethylation of DTG via GtmA is an important factor in negative regulation of gliotoxin biosynthesis in *A. fumigatus* (Dolan et al., 2014), it is not required for gliotoxin self-protection, although disruption of the SAM/SAH has been associated with gliotoxin sensitivity (Owens et al., 2015). *A. fumigatus* Δ*gliT*, which demonstrates severe sensitivity to exogenous gliotoxin, exhibited decreased levels of SAM and increased levels of SAH. Absence of GliT results in increased GtmA activity due to increased availability of DTG, increased production of BmGT and a concurrent shift in SAM/SAH ratio (Owens et al., 2015). *A. fumigatus* Δ*gliK*, which is also sensitive to gliotoxin and has attenuated GliT induction following exogenous gliotoxin exposure, also displayed altered SAM/SAH ratio in a similar manner to Δ*gliT* (Owens et al., 2015). GliT therefore plays an important role in maintaining SAM/SAH homeostasis following exposure to exogenous gliotoxin in *A. fumigatus* and absence of GliT leads to decreased SAM/SAH ratio, which contributes to gliotoxin toxicity in Δ*gliT* and Δ*gliK* (Owens et al., 2015). Furthermore, quantitative proteomic analysis of *A. fumigatus*Δ*gliK*, following exposure to exogenous gliotoxin, detected increased abundance of the Methyl/Met cycle enzymes, cobalamin-independent methionine synthase MetH/D, and methylene tetrahydrofolate reductase MTHFR/MtrA. Altered SAM/SAH was also observed in *A. fumigatus*Δ*gliK* upon gliotoxin exposure. These observations clearly inferred Met depletion consequent to *gliK* deletion and gliotoxin presence, possibly due to the observed intracellular accumulation of the *bis*-glutathionylated intermediate **2** (Figure 3). Thus, a number of unforeseen interactions are emerging between primary and so-called secondary metabolism, which may result in new anti-fungal drug target identification. Since SAM is essential for many cellular transmethylation reactions (e.g., epigenetic regulation) (Strauss and Reyes-Dominguez, 2011), and SAH may be an inhibitor of cellular methyltransferases (Burgos et al., 2012), there may be significant antifungal drug target potential associated with the overproduction of BmGT or inhibition of GliK activity- both of which may cause SAM dissipation. In addition, gliotoxin, or specific reactions required for its biosynthesis, have also been shown to influence the formation of other sulfur-containing metabolites, like EGT (Gallagher et al., 2012; Sheridan et al., 2016), and finally, the activity of gliotoxin against fungi is revealing even further interactions within biological systems- most recently involving zinc homeostasis (Vicentefranqueira et al., 2018).

### Regulation of Sulfur Metabolites by Zinc Starvation

Zn—S interactions are essential to maintain the structure of protein domains involved in protein–protein and protein–DNA interactions, to modulate catalysis in zinc-dependent enzymes and to generate redox switches in proteins involved in regulating the mobility, transfer, redistribution and sensing of cellular zinc (Maret, 2004). Indeed, the importance of Zn—S interactions for cell biology leads us to consider the existence of intricate transcriptional regulatory networks to coordinate sulfur metabolism and zinc homeostasis, particularly under zinc-limiting conditions. Transcriptomic analyses carried out to ascertain the homeostatic and adaptive response to zinc starvation in *S. cerevisiae* (Lyons et al., 2000; Wu et al., 2008) and in the filamentous fungal pathogen *A. fumigatus* (Vicentefranqueira et al., 2018), both grown in zinc-limiting versus zinc-replete media with sulfate as only source of sulfur, have revealed that zinc starvation negatively influenced sulfate assimilation. In *S. cerevisiae*, the expression of *MET3, MET14*, and *MET16*, which encode the enzymes that catalyze the first three reactions for sulfate assimilation, is repressed under zinc starvation (Wu et al., 2009). However, the expression of these genes is repressed indirectly by Zap1, which is the transcriptional regulator of zinc homeostasis in *S. cerevisiae*, because it induces the expression of *MET30* (Wu et al., 2009), which encodes an F-box protein of the SCF^Met30^ complex involved in the inactivation of the Met4 transcription factor that induces directly the expression of *MET3, MET14*, and *MET16* when the Met or SAM level is low (Rouillon et al., 2000). In *A. fumigatus* the expression level of *metR*, the ortholog of *S. cerevisiae MET4*, is slightly reduced under zinc starvation (Vicentefranqueira et al., 2018). In addition, the expression level of *cysB*, the ortholog of *MCY1* in *S. cerevisiae* that encodes a mitochondrial cysteine synthase, is also slightly reduced under zinc starvation (Vicentefranqueira et al., 2018). As noted above, the MetR transcription factor specifically induces genes required for sulfate assimilation in *A. fumigatus* (Amich et al., 2013), whereas the mitochondrial cysteine synthase CysB catalyzes the incorporation of S^2–^ coming from sulfate reduction into *O*-acetylserine to generate Cys (Brzywczy et al., 2007). Currently, it is unclear whether *metR* and *cysB* repression is exerted directly or indirectly by ZafA, which is the major transcriptional regulator of zinc homeostasis in *A. fumigatus* (Vicentefranqueira et al., 2018, 2019), although the presence of two ZR-like motifs (i.e., **Z**inc **R**esponse consensus motifs) at 2000 and 1272 bp upstream the translation start codon (TSC) of *metR* and a ZR-like motif at 318 bp upstream the TSC of *cysB* indicates that a direct ZafA-mediated repression of these genes is possible. In either case, the lower expression of *metR* and *cysB* under zinc-limiting rather than under zinc-replete conditions suggest that zinc starvation might reduce sulfate assimilation and mitochondrial Cys biosynthesis in *A. fumigatus*. In addition, the two genes more highly induced by zinc starvation in *A. fumigatus* (i.e., AFUA_7G06790/*yctA* and AFUA_4G03930/*cysX*) (Vicentefranqueira et al., 2018), exhibit a significant similarity with proteins involved in sulfur metabolism. The YctA protein of *A. fumigatus* is similar to the yeast Yct1 protein, which seems to function as a Cys transporter located in the endoplasmic reticulum (Kaur and Bachhawat, 2007), whereas the CysX protein, which has no yeast ortholog, and is predicted to be located in peroxisomes, shows a limited similarity with the CysB of *A. fumigatus* (*E*-value = 5 × 10^–9^). However, to date the role of these genes in sulfur metabolism under zinc starvation is unknown.

Besides the enigmatic effect of zinc starvation on sulfur metabolism, a distinctive feature of the adaptive response to zinc starvation displayed by *A. fumigatus* is the strong ZafA-mediated induction of the genes *gliA*, *gliT*, *gliZ*, and *gtmA* (Vicentefranqueira et al., 2018) that are involved in the biosynthesis and regulation of, and tolerance to, gliotoxin (Dolan et al., 2015). The noxious effects of gliotoxin on animal cellular physiology depends on the redox state of the two *sulfydryl* groups involved in the formation of its internal disulfide bridge (Gardiner et al., 2005). In this regard, reduced gliotoxin or DTG, which may be the most toxic form of gliotoxin, binds zinc with such high affinity that is able to remove Zn^2+^ ions from certain metalloproteins leading to their inactivation (Saleh et al., 2018). Hence, it is highly relevant that expression of *gli* genes is tightly regulated under zinc-limiting conditions.

The major regulator of gliotoxin biosynthesis is the GliZ zinc binuclear (Zn_2_Cys_6_) transcription factor (Bok et al., 2006). Although it has not been formally shown, it is very likely that GliZ induces gene expression through binding to the TCRGHNDCCGW quasi-palindromic motif (R = A or G; H = A or C or T; D = A or G or T; W = A or T), which is present in the promoter region of *gtmA* and all *gli* genes, except *gliZ* and *gliA*, and is very similar to the TCGGNNNCCGA sequence reported previously by others investigators (Fox et al., 2008). In addition, although it is unknown whether GliZ induces the expression of its own encoding gene and whether the transcription activation activity of GliZ is influenced directly by DTG and/or gliotoxin, it is well known that gliotoxin (including that of exogenous origin) enhances its own biosynthesis (Cramer et al., 2006). Interestingly, the *gliT* expression level is only slightly reduced in a Δ*gliZ* strain in the presence of exogenous gliotoxin, whereas the expression level of *gliA* reduces dramatically in a Δ*gliZ* strain following exposure to exogenous gliotoxin (Schrettl et al., 2010), despite the fact that *gliA* lacks putative GliZ binding motifs in its promoter region. In addition, *gliA* and *gliZ* are not expressed in *A. fumigatus* Δ*gliP* (Cramer et al., 2006). Therefore, in addition to GliZ, other transcription factors must influence *gliT* expression in a GliZ-independent manner, whereas *gliA* expression must depend directly on one or more transcription factors other than GliZ, provided that GliZ enhances the expression of the *gli* genes required exclusively for gliotoxin biosynthesis rather than for efflux, which is the most likely function of GliA (Wang et al., 2014). In this regard, it has been reported recently that the ZafA transcription factor, under zinc-limiting conditions, induces the expression of *gliA*, *gliT* and *gliZ*, whereby the induction level of *gliA* was >100-fold higher than that of *gliT* or *gliZ* (Vicentefranqueira et al., 2018). The promoter regions of all *gli* genes in the *gli* cluster of *A. fumigatus* (strain Af293) harbor just one ZR consensus motif to which ZafA can bind and five ZR-like motifs (i.e., **Z**inc **R**esponse consensus motifs that have one or two substitutions out of the conserved core sequence) (Vicentefranqueira et al., 2018). Interestingly, the only ZR consensus motif plus one ZR-like motif are located in the *gliZ* promoter at 822 and 776 bp, respectively, upstream of its TSC. In the promoter of both *gliM* and *gliT* there is a ZR-like motif located respectively at 124 and 483 bp upstream of the TSC, whereas in the *gliA* promoter there are two ZR-like motifs located at 455 and 508 bp upstream of its TSC (Vicentefranqueira et al., 2018). It is important to point out that the AFUA_6G09730/*gliF*-AFUA_6G09740/*gliT* intergenic sequence, which presumably functions as a divergent promoter, is only 302 bp long as annotated in the Af293 genome database. Therefore, the only ZR motif of *gliT* would not be located in the predicted *gliF-gliT* divergent promoter region but within the putative coding sequence of *gliF* (i.e., 181 bp downstream of its predicted translation start site). However, this finding raises questions about both the putative (negative) influence of ZafA on *gliF* transcription under zinc-limiting conditions and the accurate prediction of the translation start site for *gliF*. In this regard, it could be possible that the actual *gliF* translation start site is located at 699 bp rather than at 303 bp from the *gliT* translation start site and that *gliF* encoded a GliF protein with 113 amino acids less than expected. On the other hand, it is interesting to note that the two ZafA-binding motifs in the *gliA* promoter are located 130 and 183 bp upstream of the binding site for the GipA C_2_H_2_ transcription factor (positioned at 316 bp upstream of the *gliA* TSC) (Schoberle et al., 2014). It has been reported that GipA induces *gliA* and *gliP* expression in the presence of GliZ, whereas only the induction of *gliA* by GliZ depends on GipA, which led the authors to hypothesize that GliZ and GipA worked together at the same binding site (Schoberle et al., 2014). However, bearing in mind that the *gliA* promoter lacks of an obvious GliZ binding motif, the correlation between *gliZ* expression and gliotoxin biosynthesis, the essential role of ZafA for *gliA* expression during zinc starvation and the close proximity of the ZafA and GipA binding sites in the *gliA* promoter, it would appear more feasible that GipA, whose activity could also be influenced by the intracellular amount of gliotoxin, plays a role as a modulator of the ZafA-mediated induction of *gliA* under zinc starvation to adjust the *gliA* expression level to the intracellular amount of gliotoxin. Lastly, it is notable that the liquid medium routinely used for induction of gliotoxin biosynthesis (i.e., Czapek-Dox) provides a zinc-limiting culture condition (Saleh et al., 2018), which strongly suggests that zinc starvation could be the primary stimulus that triggers the ZafA-mediated induction of *gliZ* expression and, hence, gliotoxin biosynthesis, whereas the function of GliZ would be dedicated to maintain steady expression levels of the *gli* genes and gliotoxin biosynthesis while zinc starvation persists. In addition, it is remarkable that the genes required for self-protection against the noxious effects of DTG and/or gliotoxin (i.e., *gliA*, *gliT*, and *gtmA*) (Dolan et al., 2015) are strongly induced under zinc starvation (Vicentefranqueira et al., 2018). However, the lack of ZR-like motifs in the *gtmA* promoter suggests that *gtmA* expression under zinc-limiting conditions must be induced indirectly by ZafA through some unknown factor. In either case, the biological rationale that underlies the relationship between biosynthesis of the sulfur metabolite gliotoxin and zinc starvation is unknown. However, the report by Saleh et al. (2018) about the capacity of DTG to eject Zn^2+^ ions from zinc-dependent metalloproteins, represents a significant finding that will allow investigation of the biological role of gliotoxin in the context of regulating zinc homeostasis which, simultaneously, will broaden the potential practical applications of this fascinating compound.

### Ergothioneine (EGT) Biosynthesis

**FIGURE 4:**
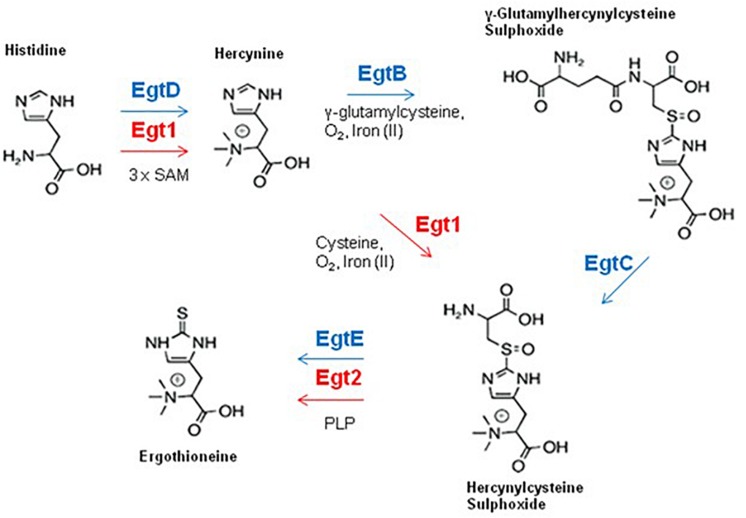
EGT biosynthesis in fungi and bacteria. Fungal enzymes are shown in red, bacterial enzymes are shown in blue. The fungal EGT pathway contains a maximum of two enzymes, uses cysteine as a sulfur source and does not produce the intermediate γ-glutamyl hercynylcysteine sulphoxide (Note Egt-1 = EgtA).

Ergothioneine (2-mercaptohistidine trimethylbetaine; EGT) is a sulfurised and tri-*N*-methylated histidine derivative, which exists in a tautomeric state between both the thione (Figure 4) and thiol forms and has demonstrable antioxidant properties. EGT exhibits a higher redox potential (*E*^0^′ = −0.06 V) than GSH (E^0^’ = 0.25 V), is less susceptible to auto-oxidation, and is more stable in aerated solutions (Hartman, 1990; Hand et al., 2005). EGT is produced by fungi (except members of the Saccharomycotina subphylum) and selected bacteria, particularly members of Actinobacterial and Cyanobacterial phyla (Jones et al., 2014). While animals or plants cannot produce EGT, they can obtain it from dietary sources or from symbiotic microorganisms (Ey et al., 2007). The seminal demonstration of EGT biosynthesis by Seebeck (2010) identified a five gene cluster (*egtA-E*) which encoded EGT production in *Mycobacterium smegmatis*. EGT biosynthesis in *M. smegmatis* is initiated by trimethylation of the NH_2_ group of histidine by EgtD, a SAM-dependant histidine methyltransferase, to yield hercynine. Hercynine is then processed by EgtB, a sulfoxide synthase, which catalyses iron(II)-dependant oxidative sulfurisation to conjugate γ-glutamylcysteine to the imidazole side chain of hercynine. The product of this sulfurisation step, γ-glutamylhercynylcysteine sulfoxide, is processed by EgtC, a glutamine amidotransferase, which cleaves the γ-glutamyl residue to yield hercynylcysteine sulfoxide. Hercynylcysteine sulfoxide is then processed by a cysteine desulfurase (EgtE) to produce EGT (Figure 4). *egtA* encodes for a γ-glutamylcysteine synthase, which is predicted to provide the substrate for EgtB mediated sulfurisation.

### EGT Biosynthesis in Fungi

The first identification of EGT in *A. fumigatus* was by Gallagher et al. (2012), who simultaneously detected EGT in a wild-type strain and increased levels of EGT in the gliotoxin null mutant, Δ*gliK*. Interestingly, *A. fumigatus* Δ*gliK* exhibited many additional phenotypes, including sensitivity to H_2_O_2_ and exogenous gliotoxin. Investigation of fungal EGT biosynthesis in *Neurospora crassa* and *Schizosaccharomyces pombe* has been undertaken by Bello et al. (2012) and Pluskal et al. (2014). The first fungal EGT biosynthetic enzyme was identified in *N. crassa* (Bello et al., 2012). Through bioinformatic analysis, they determined the gene NCU04343 contain domains from both bacterial EgtB and EgtD, which was consistent with previous observations of a fusion gene in fungal homologs of EGT biosynthetic genes (Seebeck, 2010). HPLC analysis determined that the *N. crassa* NCU04343 mutant was incapable of producing EGT. NCU04343 was thus determined to be the first biosynthetic gene in EGT biosynthesis and was renamed *egt-1*. Pluskal et al. (2014) demonstrated that deletion of the *egt-1* homolog in *S. pombe*, *egt1*, also abrogated EGT production. This was further confirmation of the importance of *egt-1* orthologs in fungal EGT biosynthesis. Ishikawa et al. (1974) demonstrated that Cys is the sulfur donor for EGT biosynthesis in fungi, as opposed to γ-glutamylcysteine utilized by bacteria (Seebeck, 2010). It has been reported that fungi do not contain orthologs of bacterial EgtA or EgtC (Jones et al., 2014), which indicates that there is no γ-glutamylcysteine synthase to produce γ-glutamylcysteine, or a glutamate amidotransferase to cleave the γ-glutamyl residue. This supports the data from Ishikawa et al. (1974) and indicates that fungal production of hercynylcysteine sulphoxide is catalyzed by *egt-1* using Cys as a sulfur donor, without the need for the intermediate γ-glutamyl hercynylcysteine sulfoxide (Figure 4).

Deletion of *A. fumigatus egtA* (AFUA_2G15650; *N. crassa egt-1* ortholog), which encodes a trimodular enzyme, completely abolished EGT biosynthesis, as determined by RP-HPLC and LC-MS/MS (Sheridan et al., 2016). *A. fumigatus*Δ*egtA* exhibited significantly reduced resistance to high levels of H_2_O_2_ and menadione, impaired gliotoxin biosynthesis/secretion, increased GSH biosynthesis, attenuated conidiation and altered cell wall integrity. EGT deficiency specifically decreased resistance to high H_2_O_2_ levels, which infers functionality as a *high-level* antioxidant (see below). Significant proteomic remodeling in Δ*egtA* was observed, compared to *A. fumigatus* wild-type, under both basal and ROS conditions, in fact, the abundance of 290 proteins was found to be altered. Of most relevance herein was the reciprocal differential abundance of cystathionine γ-synthase and β-lyase, respectively. This observation led to the conclusion that cystathionine availability could be controlled to affect EGT biosynthesis in *A. fumigatus*. As a sulfur-containing metabolite, EGT therefore plays a key role in redox homeostasis and requires essential consideration in future oxidative stress studies in *A. fumigatus*.

### EGT Function in *N. crassa* and *S. pombe*

Bello et al. (2012, 2014) used a Δ*egt-1* mutant to investigate EGT functionality in *N. crassa*. Analysis of conidial germination and viability following exposure to cupric oxide, menadione and UV light (254 nm) showed no significant differences between the wild-type and Δ*egt-1* strains. EGT was also demonstrated to play no role in protecting mycelia from cupric oxide toxicity. However, Δ*egt-1* conidia showed a significant reduction in germination compared to wild-type when exposed to tert-butyl hydroperoxide. EGT was also demonstrated to contribute to *N. crassa* conidial longevity. Furthermore, *N. crassa* Δ*egt-1* produced significantly less conidia compared to wild-type. EGT therefore appears to play an important role in conidiation and conidial health in *N. crassa*. However, no role for EGT in the mycelia of *N. crassa* was detected. Pluskal et al. (2014) generated an EGT null mutant (Δ*egt1*) and a strain producing trace amounts of EGT (Δ*egt2*) in *S. pombe*. Phenotypic analysis of these strains revealed no differences between the mutants and wild-type. Thus, EGT does not appear to play any role in protecting *S. pombe* against H_2_O_2_ and tert-butyl hydroperoxide. Metabolic analysis revealed no changes in the levels of metabolites in either strains, except for EGT, and hercynylcysteine sulphoxide in Δ*egt2*. Thus, the function of EGT in *S. pombe* is currently unclear. Bello et al. (2012, 2014) demonstrated that EGT only functions as an antioxidant in conidia, suggesting EGT function is required at specific stages of the *N. crassa* life cycle.

### The Role of EGT in *A. fumigatus* Oxidative Stress Defense and Redox Homeostasis

The availability of an *A. fumigatus* strain with abrogated EGT production allowed functional analysis of EGT in *A. fumigatus* (Sheridan et al., 2016). Sensitivity assays using ROS inducing agents with ATCC26933, Δ*egtA*^26933^ and a complemented *egtA* strain (*egtA*^C26933^) demonstrated that absence of EGT led to sensitivity to 3 mM H_2_O_2_, but not at 1 mM or 2 mM. Similarly, absence of EGT led to sensitivity to 40 and 60 μM menadione, but not 20 μM. The role of EGT for dissipating ROS was further underlined by fluorescent microscopy, which demonstrated elevated ROS levels in Δ*egtA*^26933^ compared to *A. fumigatus* wild-type, when exposed to 3 mM H_2_O_2_. Thus EGT was demonstrated to function as an antioxidant in *A. fumigatus* and to form an important component of the oxidative stress defense system in *A. fumigatus*. This was further emphasized by the demonstration that *egtA* expression was induced by 3 mM H_2_O_2._ However, it was observed that instead of resulting in increased intracellular EGT levels, it instead replenished EGT as it was dissipated in the process of detoxifying H_2_O_2_, consistent with data from Servillo et al. (2015).

Quantitative proteomic analysis demonstrated differential abundance of a multitude of reductases, oxidases, stress response proteins and enzymes with oxidizing products in Δ*egtA*^26933^ compared to *A. fumigatus* wild-type under both basal and ROS conditions (Sheridan et al., 2016). This was indicative of a shift in redox state due to EGT absence, particularly following exposure to 3 mM H_2_O_2_. Of particular importance was HapC, the redox sensitive subunit of the CCAAT-Binding Complex (CBC; Hortschansky et al., 2017). The CBC senses changes in the redox state of the cell through modification of thiol groups within the histone fold motif of HapC. It then regulates Yap1 expression to coordinate oxidative stress response (Lessing et al., 2007). HapC was demonstrated to be absent in *A. fumigatus* wild-type under ROS conditions compared to under basal conditions, indicating HapC absence is a result of elevated ROS. HapC was also absent in Δ*egtA*^26933^ compared to *A. fumigatus* wild-type under basal conditions, which suggests that Δ*egtA*^26933^ undergoes oxidative stress, even in the absence of a ROS inducing agent. This demonstrates that EGT absence leads to a significant shift in fungal redox state. EGT therefore appears to play an important role in detoxifying high levels of ROS, making it an “antioxidant of last resort.” At high levels of ROS, when these other components of the oxidative stress defense system have been exhausted or overwhelmed, EGT is then available to dissipate the remaining ROS. Absence of *egtA* further exacerbated observed redox sensitivity of *A. fumigatus* Δ*yap1* (Δ*egtA*Δ*yap1*) and fungal susceptibility to H_2_O_2_ (0.4 mM), paraquat (1.5 mM PQ), menadione (10 μM MD), *tert*-butylhydroperoxide (TBHP), iron, zinc, copper and cobalt exposure, was increased. To our knowledge, this represents a new concept in control of fungal redox homeostasis, and has received supportive commentary by comparison to EGT functionality in *Mycobacterium tuberculosis* (Cumming et al., 2018).

The characterization of EGT as an antioxidant in *A. fumigatus* offered insight into the observed rise in EGT levels in the gliotoxin-deficient mutant Δ*gliK* (Gallagher et al., 2012). In addition to increased levels of EGT, Δ*gliK* was demonstrated to exhibit significant sensitivity to H_2_O_2_ compared to wild-type (Gallagher et al., 2012), and it is speculated that it may be due to interruption of the gliotoxin biosynthetic pathway following the incorporation of GSH, as GliK catalyzes the enzymatic step directly following GliG-mediated GSH conjugation (Figure 3) (Davis et al., 2011). Thus, *gliK* deletion may result in a “GSH sink,” as GSH is committed to the defective gliotoxin biosynthetic pathway, resulting in compromised intracellular GSH content. EGT levels may therefore be increased in order to compensate for attenuated GSH levels in Δ*gliK* which would indicate a key role for maintaining redox homeostasis following *gliK* deletion. Indeed, despite attempts to generate a Δ*egtA*Δ*gliK* mutant on multiple occasions, using a variety of strategies, no successful Δ*egtA*Δ*gliK* mutant was generated (unpublished observations), which indicates that the simultaneous absence of EgtA and GliK is not possible in *A. fumigatus*. Given the observed rise in EGT levels in Δ*gliK*, it is predicted that EGT is essential to maintain redox homeostasis when *gliK* is deleted and thus a Δ*egtA*Δ*gliK* mutant may not be viable.

### Metabolic and Redox Interactions Between EGT, GSH and Gliotoxin

LC-MS/MS analysis of *A. fumigatus* wild-type, Δ*egtA*^26933^ and *egtA*^C26933^ revealed a significant increase in total GSH in Δ*egtA*^26933^ compared to ATCC26933 and *egtA*^C26933^, leading to speculation that this may be a compensatory measure to deal with the absence of EGT (Sheridan et al., 2016). Cystathionine γ-synthase (CGS; AFUA_7G01590) was undetectable by LFQ proteomic analysis in Δ*egtA*^26933^ compared to *A. fumigatus* wild-type under basal conditions. Since CGS catalyzes the conversion of Cys to cystathionine, its absence would be expected to increase Cys levels, which in turn could increase its availability for GSH biosynthesis. Concurrently, RP-HPLC and LC-MS/MS analysis revealed a significant reduction in gliotoxin levels in Δ*egtA*^26933^ compared to *A. fumigatus* wild-type. Related to this, quantitative proteomics showed that the gliotoxin oxidoreductase, GliT (Schrettl et al., 2010; Scharf et al., 2010), was undetectable in Δ*egtA*^26933^ compared to *A. fumigatus* wild-type under basal conditions. GliT catalyzes disulfide bridge closure in *A. fumigatus* and has been demonstrated to be essential for gliotoxin biosynthesis (Figure 3) (Schrettl et al., 2010; Scharf et al., 2010). Thus, decreased gliotoxin levels in Δ*egtA*^26933^ may be caused by absence of GliT. As noted, GliT has also been demonstrated to play a key role in self-protection from gliotoxin (Schrettl et al., 2010) and its absence in Δ*egtA*^26933^ might be expected to result in sensitivity to gliotoxin. However, sensitivity assays for gliotoxin revealed no significant sensitivity in Δ*egtA*^26933^ compared to *A. fumigatus* wild-type. This was explained by the observation that GliT expression was induced by gliotoxin exposure, a response that been demonstrated previously in wild-type and considered part of the self-protection strategy in *A. fumigatus.* Relevantly, *gliT* is regulated independently of the rest of the *gli* cluster, which allows gliotoxin biosynthesis and self-protection to function autonomously (Schrettl et al., 2010).

**FIGURE 5:**
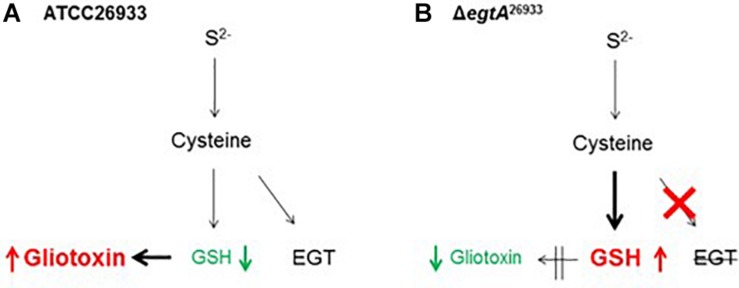
Proposed interaction between EGT, GSH and gliotoxin biosynthesis in *A. fumigatus*. **(A)** In *A. fumigatus* wild-type (ATCC26933), high levels of gliotoxin biosynthesis result in reduced levels of intracellular GSH due to the double glutathiolation step in gliotoxin biosynthesis. This is facilitated by EGT production which compensates for decreased levels of GSH, to maintain cellular redox homeostasis. **(B)** In Δ*egtA*^26933^, absence of EGT results in a reduction in antioxidant potential or buffering against ROS, necessitating increased levels of GSH to compensate. GSH is thus diverted from gliotoxin biosynthesis, resulting in decreased levels of gliotoxin.

Gliotoxin and GSH metabolism are intrinsically linked because GSH is essential for gliotoxin biosynthesis. As noted, Davis et al. (2011) and Scharf et al. (2011) showed that GSH is incorporated into the gliotoxin biosynthetic pathway via GliG. GSH is the thiol source for gliotoxin, which is essential for its function, and deletion of *gliG* results in the complete abrogation of gliotoxin biosynthesis (Figure 3). Thus, the observed increase in GSH levels and concurrent decrease in gliotoxin levels in Δ*egtA*^26933^ could be related. Gliotoxin biosynthesis may be curtailed in order to diminish GSH incorporation into the gliotoxin biosynthetic pathway, thereby allowing for an increase in intracellular GSH. EGT therefore appears to be an important factor in gliotoxin production, as it compensates for reduced levels of intracellular GSH which is required for gliotoxin biosynthesis. In the absence of the antioxidant potential of EGT, GSH must be diverted from gliotoxin biosynthesis, resulting in reduced levels of gliotoxin. It may therefore be the case that EGT is required to maintain the optimal cellular redox state for high levels of gliotoxin production (Sheridan et al., 2016). This was further emphasized by results for total GSH measurement in *A. fumigatus* AfS77 and Δ*egtA*^AfS77^. In contrast to *A. fumigatus* ATCC26933, deletion of *egtA* in the AfS77 background resulted in no significant changes in GSH levels. However, it was particularly interesting that GSH levels in AfS77 were significantly increased compared to ATCC26933. AfS77 is derived from ATCC46645 (Hartmann et al., 2010), which has been demonstrated to produce low levels of gliotoxin compared to ATCC26933 (Schrettl et al., 2010). Thus, in ATCC26933, decreased levels of GSH compared to AfS77 may be a result of increased gliotoxin biosynthesis, relative to AfS77. In ATCC26933, EGT is required for high level gliotoxin production, due to the necessary utilization of GSH. When *egtA* is deleted and no EGT is present, a metabolic adjustment occurs whereby gliotoxin biosynthesis is decreased in order to increase GSH levels (Figure 5). Notably, the levels of GSH in Δ*egtA*^26933^ were similar to those of AfS77 and Δ*egtA*^AfS77^ (i.e., there was no significant differences in the levels of GSH in Δ*egtA*^26933^, AfS77 and Δ*egtA*^AfS77^). An increase in GSH levels was not observed in Δ*egtA*^AfS77^ compared to AfS77, because low levels of gliotoxin production in AfS77 results in higher basal levels of GSH compared to ATCC26933. This meant that a metabolic adjustment was not required in the absence of EGT as GSH levels were already sufficient.

**FIGURE 6:**
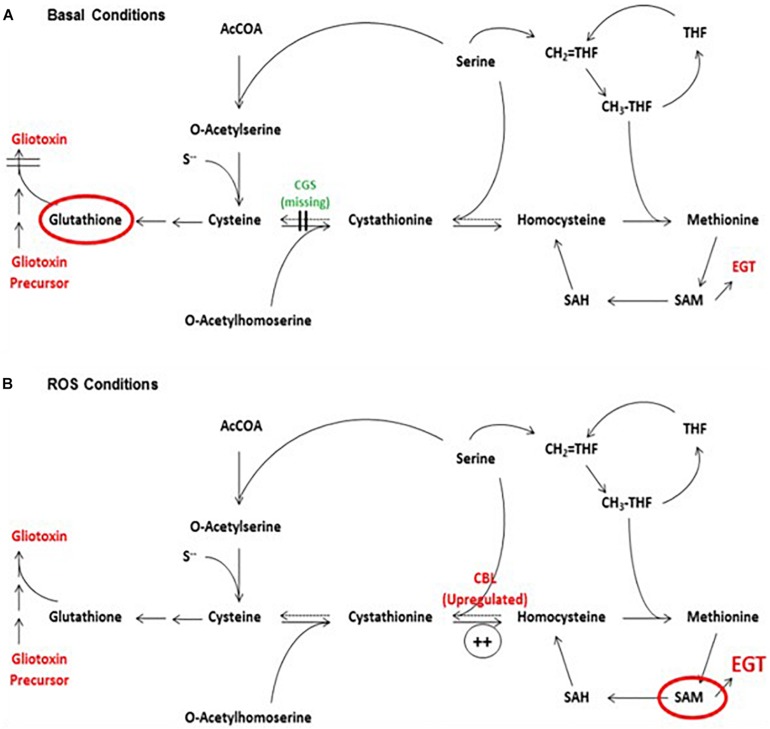
The proposed “cystathionine switch” in *A. fumigatus*. **(A)** Comparison of Δ*egtA*^26933^ and *A. fumigatus* wild-type under basal conditions reveals the absence (undetectable) of cystathionine γ-synthase (CGS), which normally converts cysteine to cystathionine. This may facilitate a switch towards increased GSH production by virtue of Cys excess. **(B)** Comparison of Δ*egtA*^26933^ and ATCC26933 upon addition of 3 mM H_2_O_2_ resulted in an increased abundance of cystathionine β-lyase (CBL), which converts cystathionine to homocysteine. This is predicted to result in increased production of SAM, which is essential for EGT biosynthesis. From Sheridan et al. (2016), under a CC BY 4.0 international license. This model predicts elevated requirement for Met and SAM under ROS conditions, which further underpins MetH and SasA essentiality in fungi.

The interplay between gliotoxin, GSH and EGT biosynthesis was also evident in Δ*gliK*. Deletion of *gliK* resulted in abrogated gliotoxin biosynthesis and increased levels of EGT (Gallagher et al., 2012). As discussed previously, this was speculated to be caused by the interruption of the gliotoxin biosynthetic pathway directly following the incorporation of GSH, resulting in a “GSH sink.” Thus, we propose that gliotoxin and EGT biosynthesis are interlinked, with a possible involvement of GSH, which infers that *A. fumigatus* must carefully balance production of gliotoxin, GSH and EGT. As a result, disruption of one biosynthetic pathway may result in alterations in the other two. All three metabolites are redox-active molecules and, as such, the levels of each may alter to maintain cellular redox homeostasis. As demonstrated in Δ*egtA*^26933^, abrogation of EGT biosynthesis resulted in a shift in the redox state of the cell, which required the alteration of gliotoxin and GSH biosynthesis to compensate (Figure 5). The biosynthetic pathways of gliotoxin, GSH are EGT are also linked metabolically, as Cys provides the thiol group for all three; directly in the case of EGT and GSH, and via GSH in the case of gliotoxin (Figure 5). Additionally, the biosynthesis of both gliotoxin and EGT contains methyltransferase steps, requiring SAM as a co-factor. Thus, Cys availability (and therefore sulfur) will impact biosynthesis of all three, while levels of SAM will affect EGT and gliotoxin biosynthesis. Production of these metabolites must therefore be balanced against the availability of Cys and SAM. SAM levels are of particular interest in EGT biosynthesis as 3 moles of SAM are required for every 1 mol of EGT produced. The importance of Cys and SAM availability for these metabolic adjustments was emphasized by the observation of a “cystathionine switch,” as demonstrated in Δ*egtA*^26933^. During quantitative proteomic analysis under basal conditions, cystathionine γ-synthase (CGS; AFUA_7G01590) was undetectable in Δ*egtA*^26933^ compared to ATCC26933, while cystathionine β-lyase (CBL; AFUA_4G03950) exhibited a log_2_ 3.1-fold increase in abundance in Δ*egtA*^26933^ compared to ATCC26933 under ROS conditions (Figure 6). This suggests that *A. fumigatus* Δ*egtA*^26933^ can switch from directing Cys toward GSH biosynthesis under basal conditions, to producing SAM to effect EgtA-catalyzed abortive His trimethylation which would ordinarily enable EGT biosynthesis under ROS conditions. Cystathionine therefore appears to be at the nexus of the metabolic response to EGT absence which infers a hitherto unknown but prominent role for cystathionine in directing the cellular metabolic thiol flux (Sheridan et al., 2016).

## Conclusion

The essential requirement for sulfur uptake and incorporation into essential biomolecules by pathogenic fungi represents a potential therapeutic *Achilles heel* – especially because many of the associated enzymatic reactions are fungal–specific. One area which requires prompt research attention is to obtain a greater understanding of the mass balance of sulfur-containing compounds in fungi under normal and stress conditions. The attractiveness of targeting fungal sulfur metabolism is further enhanced considering emerging links between hitherto cryptic and often apparently distinct biosynthetic pathways, such as those involving SAM/SAH homeostasis, gliotoxin, related ETPs and EGT. In addition, interference with nascent fungal zinc–sulfur homeostasis systems represents an even more recent potential target to enable antifungal drug target identification, as does the intricate interaction between redox-active fungal metabolites as exemplified via an apparent ROS-dependent cystathionine switch mechanism in *A. fumigatus*. A specific list of targets could include direct interference with fungal sulfur uptake via transport inhibitors, blocking EGT biosynthesis via EgtA inhibition (along with Yap1 inhibition), impeding specific steps in ETP biosynthesis (e.g., GliK), inhibition of pantothenic acid, Met and/or Cys biosynthesis or SAH degradation and finally small-molecule interference with enzymes involved in cystathionine formation in fungi. Without a doubt, interfering with fungal sulfur metabolism has many more surprises and opportunities in store for us.

## Author Contributions

AT, KS, GJ, JC, and SD wrote, edited, and approved the manuscript.

## Conflict of Interest

The authors declare that the research was conducted in the absence of any commercial or financial relationships that could be construed as a potential conflict of interest.
